# Innovative 3D and 2D machine vision methods for analysis of plants and crops in the field

**DOI:** 10.1016/j.compind.2018.02.002

**Published:** 2018-05

**Authors:** Lyndon Neal Smith, Wenhao Zhang, Mark F. Hansen, Ian John Hales, Melvyn Lionel Smith

**Affiliations:** Centre for Machine Vision, Bristol Robotics Laboratory, University of the West of England, Bristol, Bristol BS16 1QY, UK

**Keywords:** 3D vision, Plant analysis, Photometric stereo

## Abstract

Machine vision systems offer great potential for automating crop control, harvesting, fruit picking, and a range of other agricultural tasks. However, most of the reported research on machine vision in agriculture involves a 2D approach, where the utility of the resulting data is often limited by effects such as parallax, perspective, occlusion and changes in background light – particularly when operating in the field. The 3D approach to plant and crop analysis described in this paper offers potential to obviate many of these difficulties by utilising the richer information that 3D data can generate. The methodologies presented, such as four-light photometric stereo, also provide advanced functionalities, such as an ability to robustly recover 3D surface texture from plants at very high resolution. This offers potential for enabling, for example, reliable detection of the meristem (the part of the plant where growth can take place), to within a few mm, for directed weeding (with all the associated cost and ecological benefits) as well as offering new capabilities for plant phenotyping. The considerable challenges associated with robust and reliable utilisation of machine vision in the field are also considered and practical solutions are described. Two projects are used to illustrate the proposed approaches: a four-light photometric stereo apparatus able to recover plant textures at high-resolution (even in direct sunlight), and a 3D system able to measure potato sizes in-the-field to an accuracy of within 10%, for extended periods and in a range of environmental conditions. The potential benefits of the proposed 3D methods are discussed, both in terms of the advanced capabilities attainable and the widespread potential uptake facilitated by their low cost.

## Highlights

• 3D and 2D vision plant and crop analysis with advanced functionality, is described.
• Challenges for robust and reliable machine vision in the field, are considered.
• Photometric stereo enables recovery of plant textures at high-resolution.
• Widespread uptake of the proposed methods is facilitated by their low cost.

## 1 Introduction

Predicted increases in world population, coupled with the effects of climate change, mean that the need for increases in the efficiency of agricultural methods, concurrent with reductions in their environmental impact, is becoming critical for ensuring sustainable production. Although food production has increased significantly over the last century, due largely to the effects of mechanisation and intensive farming methods, this has come at the cost of an increased utilisation of resources such as water, fertilizers, herbicides and pesticides, which is unsustainable for meeting future demands, both economically and ecologically. It is then perhaps fortuitous that advanced technologies are now emerging that offer potential for providing plant-related data and knowledge that can enable increases in productivity while reducing environmental impacts. This can be realised in the form of new generations of autonomous agricultural robotic devices that will operate with the needed levels of information and intelligence. These could take the form of, for example, small tractors fitted with sensor systems that enable them to operate autonomously and to identify features of interest – such as weeds. Upon detection a weed could then be destroyed by a method such as use of a servo system for spraying a small amount of herbicide at its meristem. Such an approach offers obvious cost and ecology benefits compared to general spraying of herbicide, as well as additional environmental benefits associated with producing less soil compaction than a full-sized tractor and use of less fuel. However, significant technical developments are needed before such a device can operate autonomously and effectively in the field; and principal among these is an effective sensor system. While instrumentation such as GPS, inclinometers and accelerometers may play important roles, perhaps the most useful sensor for agricultural automation is that of machine vision. In fact it could be argued that this is a critical enabling technology, and that significant breakthroughs in machine vision will enable dramatic increases in the use of agricultural automation/robotics. In recent decades there has been considerable development of 2D machine vision technologies for plant detection/analysis; however there has only been very limited transfer of these technologies to use in the field/outdoors. Reasons for this include complications resulting from variations in illumination experienced outdoors, as well as the complexities of images captured. Difficulties can occur in image interpretation/analysis and feature recognition/measurement, due to the inherent complexities of plant morphologies (and therefore the required complexities of the associated models) with factors such as unexpected occlusion of view and/or shadowing. If methods could be found for alleviating these difficulties, then the effective employment of machine vision for plant analysis, and all the associated benefits mentioned above, could be realised. One such method is that of 3D machine vision; where, for example, leaf surfaces can be recovered in 3D, so that the true area can be calculated (rather than its projection in a particular direction); also, the 3D information on the leaf allows its occlusion or shadowing effects to be evaluated, thereby assisting with interpretation of conventional images of the plant. The utility of 3D machine vision in agriculture does in fact go well beyond simply assisting with understanding the general shape and structure of a plant. Depending upon the exact nature of the 3D machine technique applied, a range of types of information can be derived that can inform the farmer, depending upon the particular needs and requirements. In this paper this range is illustrated by two very different case studies of 3D vision systems that provide useful information in the field. The first describes a 3D machine vision technique that has undergone extensive development in the Centre for Machine Vision (CMV) at the University of the West of England (UWE) – photometric stereo (PS); and goes on to show how this can utilize off-the-shelf components in novel configurations that can generate high-resolution 3D surface data for facilitating directed weed elimination (as described above), through to new types of plant phenotyping. The second employs existing low-cost high-performance 3D vision systems and shows how they can be combined with GPS data for providing useful produce and field information during potato harvesting.

## 2 Related work

A great deal of literature exists that reports on studies of computer vision agricultural applications; and currently a growing amount is specifically addressing 3D vision work. There is not space here to review all of this; rather, a selection of work will be reviewed which is particularly relevant to the subject of this paper and the associated research. In 2016 Vázquez-Arellano et al. [1] completed a comprehensive review of 3D Imaging Systems for Agricultural Applications. They identified reduced labour availability, scarcity of natural resources, and consumer demand for quality products as drivers for automation in agriculture; and stated that 3D vision is a key technology for agricultural automation. In their 2010 paper [2], McCarthy et al. state that field environment precision agriculture applications face the challenge of overcoming image variation caused by the diurnal and seasonal variation of sunlight; and put forward a view that augmenting a monocular RGB vision system with additional sensing techniques potentially reduces image analysis complexity while enhancing system robustness to environmental variables. We suggest that 3D data recovery comprises one such additional sensing technique. In their 2015 review of sensors and systems for fruit detection and localisation [3], Gongal et al. identify occlusions, clustering, and variable lighting conditions as the major challenges for the accurate detection and localization of fruit in the field environment. They say that improved accuracy can be achieved through 3D fruit localisation, but point out that methods such as laser range finding are currently bulky, slow and costly – however these drawbacks do not apply to other 3D vision approaches, such as a RGB-D camera or photometric stereo. In 2017 Binch and Fox reported on an interesting controlled comparison of machine vision algorithms for *Rumex* and *Artica* detection in grassland [4]. They conclude that all the accuracies in their implementations were lower than those of other researchers and suggest a number of reasons for this, which were characteristic of their collecting real data in the field – for example, they required their data to come from a wide mixture of lighting and weather conditions. This provides further evidence of the need to address such factors in order to collect data in the field that will be of real use to farmers. They also conclude that: “…the best performing method for the overall spray/no spray decision is based upon Linear Binary Patterns with Support Vector Machine classification…” – this is in line with our findings in the same area and will form part of a future CMV paper. (Although we have also found that convolutional neural networks provide advanced capabilities for weed identification in complex grassland images, as outlined below.) The demands of in-the-field operation are further illustrated by the example of using machine vision for analysing potato harvesting. Some previous work has been reported on vision inspection for potatoes [[5], [6], [7]] and in 2015 Rady provided a review of Rapid and/or non-destructive quality evaluation methods for potatoes [8]. However, the systems described in these papers generally operate indoors and often in laboratory type conditions (i.e. with careful control of lighting and viewing directions). In contrast to this, there are many significant challenges associated with real-time in-the-field analysis of potato harvesting production (and in fact all in-the-field crop/plant analysis machine vision applications); and these are described in Section 4.2.1. The extent to which these can be successfully addressed is highly dependent upon the manner in which images are captured and how the 2D/3D data are generated – particularly what type of camera is employed and how it is configured. Two types of cameras/imaging were employed in the current work: specifically a RGB-D device and photometric stereo – therefore some discussion is provided below of these devices/techniques.

## 3 Cameras and techniques employed for imaging in-the-field

### 3.1 The RGB-D camera

RGB-D cameras such as the Microsoft Kinect have been increasingly employed for machine vision research (particularly 3D work) due to their common availability, low cost and relatively good range-finding performance. For example, the RGB-D camera employed in the current work captured data at a rate of 30 frames per second; and for each frame that was captured by the camera, a 2D colour image and a depth map were generated. The latter gives a distance, in mm from the camera, for each pixel, thereby generating a 3D point-cloud. Two versions of RGB-D camera were experimented with; the first employs a distortion of a projected infra-red pattern to calculate the depth data, while the second uses time-of-flight technology. However, the limitation in the pattern (and camera) resolution, in combination with the fixed field of view and ranges which these devices operate at, and the limitations of the USB interfaces they employ, mean that the 3D data obtained are of relatively low resolution when compared to the dense arrays of surface normals that can be captured using photometric stereo (see below). Despite this there are some agricultural applications where the RGB-D range data can prove useful – an example is provided by the potato measurement application described in Section 4.2. However, the analysis of individual plants, recovering features such as true leaf colour/area and leaf veins, for monitoring plant growth and phenotyping, is beyond the capability of the RGB-D device, but can be achieved with a photometric stereo analysis.

### 3.2 Photometric stereo

**Fig. 1:**
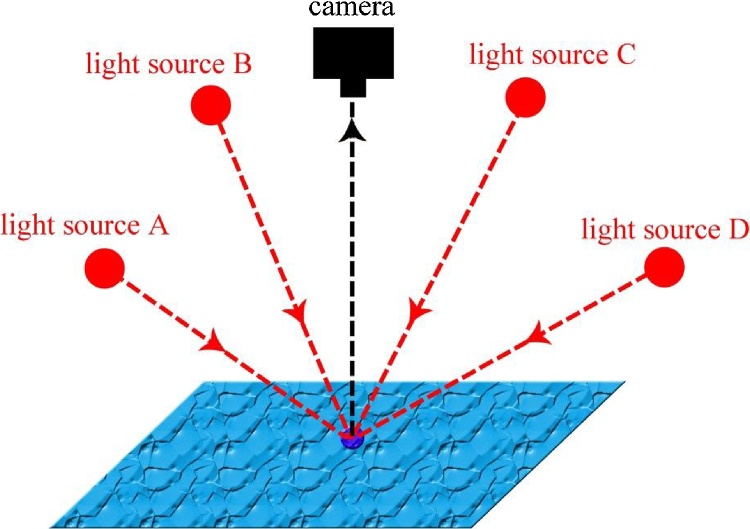
The principle of photometric stereo, which employs a single camera to capture multiple images of a surface illuminated by multiple light sources.

Photometric stereo (PS) is a technique first described by Woodham in 1980 [9], which employs a single camera and a set of at least 3 lights in known locations. Here, rather than calculating a depth image or a point cloud, a surface normal field is recovered from an object that is illuminated from different directions while the viewing direction is held constant (see Fig. 1). The fraction of the incident illumination reflected in a particular direction is dependent on the surface orientation, which can be modelled using *Lambert’s Law* (this model assumes that light is reflected uniformly in all directions from a surface). Therefore, when the directions of incident illumination are known and the radiance values are recorded, the surface orientation can then be derived.

Woodham observed that three views are sufficient to uniquely determine the surface normals as well as albedos at each image point, provided that the directions of incident illumination are not collinear in azimuth. Four illuminants/views can be employed for improved reconstruction performance.

The equations involved in determining the albedo and surface normal vectors from the three recovered images can be derived:

Let $I_{1}\left(x,y\right)$, $I_{2}\left(x,y\right)$ and $I_{3}\left(x,y\right)$ be the three images captured under varied illumination directions. By varying the illumination direction, the reflectance map is changed accordingly, giving Eq. (3.1).

(3.1) $$\{\begin{array}{c} I_{1}\left(x,y\right)=R_{1}\left(p,q\right)\\ I_{2}\left(x,y\right)=R_{2}\left(p,q\right)\\ I_{3}\left(x,y\right)=R_{3}\left(p,q\right) \end{array}$$

where $R_{1}\left(p,q\right)$, $R_{2}\left(p,q\right)$ and $R_{3}\left(p,q\right)$ are the reflectance maps under different illumination directions, while p and q are gradients of the surface in the x and y directions, respectively. A general reflectance map in the gradient representation of the surface orientation and illumination direction is expressed in Eq. (3.2) [9].

(3.2) $$R\left(p,q\right)=\frac{\varrho \left(1+pp_{s}+qq_{s}\right)}{\sqrt{1+p^{2}+q^{2}}\sqrt{1+{p_{s}}^{2}+{q_{s}}^{2}}}$$

where *ϱ* is the albedo, $\vec{N}=\left[-p,-q,1\right]$ defines the surface normal vector, and $\vec{L}=\left[-p_{s},-q_{s},1\right]$ defines the illumination direction. Let the surface be z=f(x,y), the gradients in *x* and *y* directions become:

(3.3) $$\{\begin{array}{c} p=-\frac{\partial f\left(x,y\right)}{\partial x}\\ q=-\frac{\partial f\left(x,y\right)}{\partial y} \end{array}$$

These equations are derived under the assumptions that 1) the object size is small relative to the viewing distance. 2) The surface is Lambertian. 3) The surface is exempt from cast-shadows or self-shadows.

The PS method reconstructs one surface normal vector per pixel, and therefore it is capable of recovering surface normals in high resolution. 3D reconstructions by PS are spatially consistent with PS images (greyscale) captured by a single camera. This eliminates the correspondence problem that places a computation burden upon binocular vision solutions, i.e. the problem of ascertaining how pixels in one image spatially correspond to those in the other image. Furthermore, the resolution of PS reconstructions is flexible and is solely determined by the camera and lens employed; thereby allowing PS to be configured for a specific device or application. In contrast, data obtained by RGB-D cameras are normally of low spatial and depth resolution, which severely degrade as the sensor-object distance increases; and such cameras are generally non user-configurable. In addition, PS reconstructions provide detailed high-frequency 3D texture information. 3D depth information can be derived from PS surface normals when necessary (although some errors can be introduced during this step). In contrast to PS, binocular stereo is more prone to noise and artefacts, since it directly recovers depth of surface (i.e. image centred) data rather than surface orientations (i.e. object centred). Although being highly accurate and of high resolution, PS devices can be constructed at a similar or lower cost to the RGB-D or Kinect camera, with the potential flexibility of being portable or long-range; and thus comprise a powerful solution to 3D imaging.

Despite these significant potential advantages, utilisation of photometric stereo in machine vision applications has been rather limited in comparison to other techniques such as the RGB-D camera mentioned above. This is perhaps because RGB-D cameras are available off the shelf at low cost in the form of devices such as the Kinect; but this is not true of photometric stereo, where instead the user is obliged to mount and configure a camera and a set of lights in known orientations. In addition, it is necessary to switch each light at high speed and in exact synchronisation with the image capture – all of which can prove to be a considerable challenge in terms of instrumentation and programming. A final reason is that the RGB-D camera, as the name suggests, concurrently produces RGB and depth images for a given scene. In contrast, implementing photometric stereo requires processing and combining image intensities and to ensure maximum resolution when doing this, grey-scale cameras are usually employed – consequently the albedos often emerge as grey-scale images. It is however possible to also capture RGB images by replacing the grey scale camera with a colour one; and we have done this in the field, for plant analysis.

Having given an outline of the capabilities of the cameras employed, the following section describes the experiences of applying our machine vision technologies for crop/plant analysis in the field.

## 4 Machine vision case studies for in the field crop/plant analysis

### 4.1 Use of machine vision for weed detection and analysis: experiments and observations

#### 4.1.1 2D weed detection and analysis in the field

**Fig. 2:**
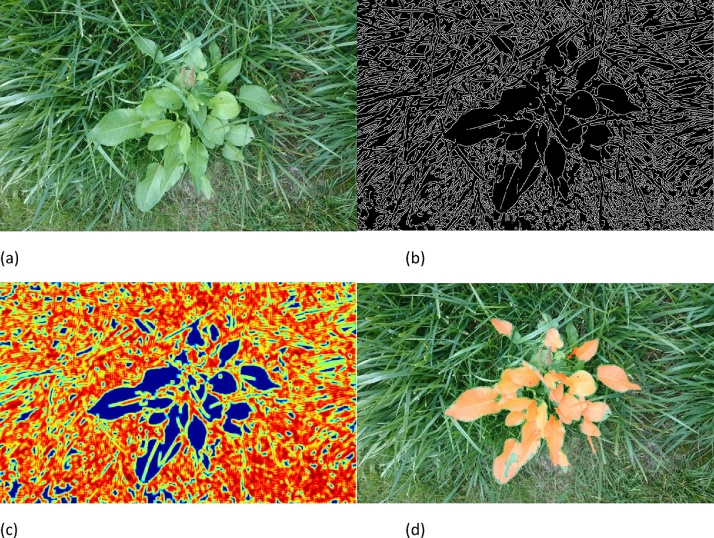
Conventional image analysis for segmentation of dock (*Rumex obtusifolius*) in grass: (a) captured image, (b) edge filtered, (c) local entropy filtering, with a 32 × 32px window – high entropy is hotter colours (red/yellow) and low entropy is cooler (blue), (d) erosion/dilation and thresholding of this entropy, shown against the original image – orange shows dock leaves have been identified. (For interpretation of the references to colour in this figure legend, the reader is referred to the web version of this article.)

Recent crop/plant machine vision work that has been undertaken by CMV in the field is that of 2D weed detection and analysis employing a conventional colour camera mounted on a tractor without any shrouding [10]. This produced data that were usefully processed for detection of weeds in grass, which is less trivial than detection of weeds on dirt – the latter can be achieved through analysis of the green and red components of images from colour cameras, for identification of greenness [11]. Our approach to separating the weeds such as dock from the grass involved: edge detection, texture analysis through use of a local entropy filter to differentiate between areas of high entropy (grass, owing to the higher frequency, multidirectional texture) and low entropy (dock leaves), and image processing techniques such as erosion and dilation/thresholding, as shown in Fig. 2.

The chief complication with this approach is associated with unexpected and dramatic changes in illumination levels. The cause of this can range from the sun emerging from behind cloud, through to the tractor changing direction, resulting in a change in brightness due to a change in the relative position of the sun, or shadows being cast by the tractor or associated equipment. The most serious potential problem associated with light level change is the possibility of image saturation. The chance of this occurring can be minimised through use of an automatic shutter speed adjustment or (if the intensity change is severe), automatic iris adjustment. The only problem with employing such approaches is that they will take a short time to adjust to the light level change and during this time good image capture may not be possible, resulting in data loss. The second potential drawback associated with light level changes and shadowing is that it introduces a level of complexity in the images which may result in simple image processing techniques being ineffective for weed feature identification/segmentation. A possible solution to this problem involves the application of machine learning to the weed segmentation. In CMV, neural networks trained on extensive sets of grass and weeds-in-grass images have shown considerable robustness to changes in illumination. Many of our data sets have been collected under conditions of dramatic changes in illumination, with shadows commonly present. The resulting complexities of the images has been exacerbated by an observed wide variation in dock sizes, with some docks being clustered and other being sparsely distributed, as well as the presence of grass/dried grass of different lengths. However, analysis of such scenes has benefitted from recent advances in open-source technologies such as TensorFlow, which have offered new opportunities to experiment freely with contemporary neural network architectures. Particularly promising results have been attained with the application of convolutional neural networks, which were found to be able to reliably identify weeds even in the presence of severe changes in image brightness. Although this approach has the disadvantage of requiring large amounts of training data and a potentially significant training time; this is a promising area of research that is currently being intensively investigated throughout the computer vision community. A high performance computer has been installed in CMV to progress this work, as well as other in-depth image analysis methods, such as Local Binary Pattern modelling with Support Vector Machine classification. In recent tests, we have repeatedly demonstrated reliable detection of dock in images of grass that were captured under a wide range of illumination conditions and when less than 5% of the image concerned was comprised of dock leaf. In addition to conventional 2D imaging, there are 3D machine vision approaches that may provide solutions to illumination change problems as well as potentially offering various types of advanced capabilities.

#### 4.1.2 3D weed detection and analysis in the field

Experiments were conducted on plant analysis using 3D data from a RGB-D camera and it was found that meristem identification was possible for some types of plant. Although the depth resolution limitations resulting from the USB2 interface for the camera used (Kinect 1, see below), meant that increased accuracy for meristem detection was not demonstrated, such increased accuracy might be attainable for future 3D measurements using the Kinect version 2 with a USB3 interface. It was however found that combining 3D range thresholding with analysis of 2D image data can improve the reliability of weed identification and enable weed segmentation and measurement of plant row separation [12]. We have also overlaid a grid on images (employing every 10th pixel) and have applied a dense optical flow method to calculate (2D or 3D) field maps. As mentioned above, recovering features such as leaf veins, for monitoring plant growth and phenotyping, is beyond the capability of the RGB-D device. This is because the Kinect 1 has a depth resolution greater than 1 mm [13], while leaf vein thicknesses are often less than 1 mm (as we have found in many of the leaf measurements we have taken in CMV). The limitation in the 3D resolution of the Kinect is due to the fact that the camera measures range and the limitation in the resolution of the projected pattern and/or bandwidth of the USB camera interface limits the resolution with which 3D features can be detected. However, PS can be usefully employed to analyse such features.

#### 4.1.3 Static four-source photometric stereo for plant analysis

In addition to recovering the texture and shape of the surface, photometric stereo provides a robust means of separating surface 3D and 2D textures – thereby providing good quality 3D surface data and accurate colour data in situations where conventional images would reflect hue effects or changes in surface pigmentation. Once the 3D surface shape data has been recovered, it needs to be analysed in order to identify meristems for implementing directed weeding (most efficient weed eradication will occur if herbicide is applied selectively to the part of the plant that is growing). To do this we experimented with various methods for surface modelling; and employed two metrics: “Shape Index”, as proposed by [14] and “HK Segmentation” [15] (based on “principal curvatures” calculated using the Hessian matrix of the second order partial differentials of the surface). The specific implementation details are beyond the scope of this paper but tests indicated that the HK-measure gives a better indication of where the meristem may fall, compared to the shape index.

**Fig. 3:**
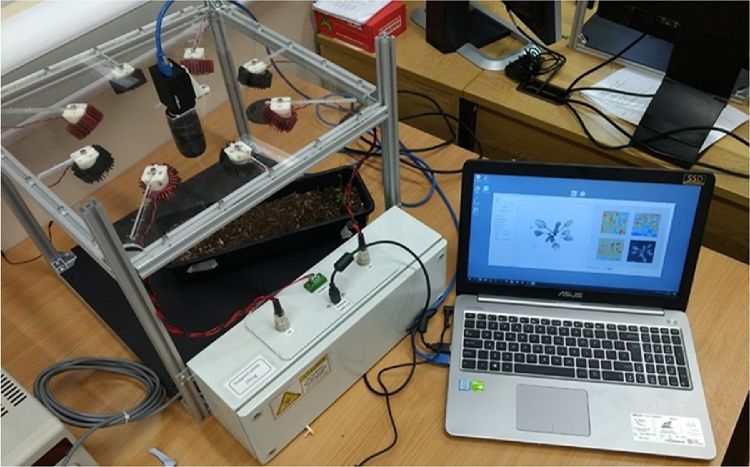
Low-cost photometric stereo rig for recovering plant 2D and 3D data to enable measurement of phenotypic traits; where two sets of four LEDs are employed for performing photometric stereo at two wavelengths – one visible and the other near-infrared.

A major advantage of photometric stereo is that the surface can be analysed in 3D at a generally much higher resolution than is the case for other methods – in fact it is only limited by the imaging resolution of the camera/lens employed. Consequently, the use of photometric stereo also offers potential to employ machine vision for generating new advanced capabilities for plant analysis such as phenotyping. Fig. 3. shows a low-cost photometric stereo rig that we have developed at CMV specifically for imaging plants (in visible and near-infrared illumination), to measure phenotypic traits.

**Fig. 4:**
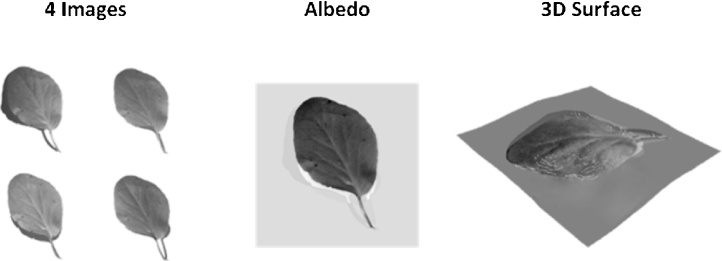
Four images captured using Fig. 3 rig, which are combined with a lighting model to generate the albedo 2D leaf data and the 3D leaf surface.

Fig. 4 shows four images of a leaf captured with the Fig. 3 rig. These are combined with the lighting model (Lambert’s Law) to recover the 2D albedo and the 3D surface. In the albedo, lighting effects are eliminated, which allows the vein structure to be more clearly identified – this is useful for plant classification/phenotyping. On the right hand side in Fig. 4, the 3D surface of the leaf has been reconstructed, so that the leaf surface information is known at each point. This allows parallax effects to be eliminated, resulting in a more accurate estimate of the leaf area, which is useful for monitoring plant growth in various growing environments/conditions. Near-infrared light sources are employed in the Fig. 3 rig, which we have found to have the advantage of light reflection being more Lambertian – thereby reducing errors in the recovery of the surface normals, as well as allowing the plants to be imaged 24 h a day over long periods without interfering with their growth cycles (these near-infrared findings are being detailed in a paper which is in preparation). We have in fact employed LEDs of wavelength 940 nm, with matching narrow-bandpass filters, to capture data similar to that shown in Fig. 4, but which were captured while the leaf was subject to direct and bright sunshine. This approach, which makes use of the natural dip in sunlight intensity at around 940 nm [16], offers great potential for capturing high-resolution 3D data from plants in greenhouse environments, or outdoors, without the need for controlling background light. We therefore consider that near-infrared photometric stereo has great potential; and it is the subject of on-going intensive research effort in CMV. Our investigations also indicate that photometric stereo can enhance the utility of hyperspectral imaging for revealing physiological and structural characteristics in plants. The spectral response of vegetation is highly affected by its orientation [17]; however this effect can be allowed for by incorporating photometric stereo surface normal data – thereby increasing the robustness of spectral measurements without increasing the overall price of the system.

#### 4.1.4 Two-source photometric stereo for plant analysis from a moving platform

A four source PS system is incapable of capturing data from a moving rig as motion between the first and last frame would prevent successful calculation of the surface normals. Therefore we instead have implemented a two-source method [18] which allows calculation of the surface gradient in one direction (2.5D). This method requires lights to be in line with the x-axis of the camera (i.e. the direction of movement) in order to recover surface normals in that plane. We developed and described application of ‘dynamic photometric stereo’ to tile inspection in 2005 [19]. Here we employed two near infra-red line lights to recover surface normals from a tile moving along a conveyor. At that time we found that although this does not allow full 3D reconstruction of the surface, the gradient along the x-direction is sufficient to obtain useful representation of surface shapes – thereby enabling segmentation of moulding defects. The situation for plant analysis is similar, in that again the surface normal data using only two sources is incomplete, but the component in the x plane can still be used for locating the meristem. We achieved this by manually labelling the meristems in a series of plant gradient maps, and then employed localised histograms of gradients for training a classifier, using techniques that included support vector machine (SVM). The classifier was then used to scan gradient maps of other plants to identify similar gradient features (with strength of match indicated by a heat–map), thereby locating a meristem. Initial tests showed that although identification of the plant meristems was more challenging than in the case of four-light PS, it could still be achieved. On-going work involves further increasing the resolution of the gradient maps, combined with more powerful neural network modelling; with the aim of increasing meristem location reliability over a wide range of plant types.

Following on from the weed detection work, the wide range of tasks for which machine vision can provide useful agricultural information is further illustrated by another application developed in CMV. Here newly emerging low-cost vision technologies are employed to gather 3D potato tuber data in the field, during harvesting.

### 4.2 Measurement of the size distribution of potatoes as they are harvested in the field

Although the potato metrology system employed off-the-shelf vision system components, its development and implementation was nevertheless non-trivial, due to the technical challenges associated with robust and reliable extended operation of a vision system outdoors (particularly in an agricultural environment). The application is concerned with reliable measurement of the size distribution of potatoes as they are harvested in the field, together with concurrent recording of GPS position data.

#### 4.2.1 Challenges associated with reliable and robust in-the-field operation

The challenges associated with this can be summarised as follows:

1. Exposure to the elements (e.g. driving rain); also, the system may be subject to cleaning by being sprayed with water from a hose.
2. Dramatic changes in background lighting, both intra- and inter-frame, ranging from direct sunlight to heavy shade.
3. The system will be mounted on a harvester which will be vibrating and moving around.
4. The power supply from the harvester/tractor may be intermittent.
5. Relevant GPS data need to be recorded in real time, associated with the 3D data capture, collated and made easily available to the user.
6. The potatoes will be moving on a conveyor within the harvester, and some will be in contact.

**Fig. 5:**
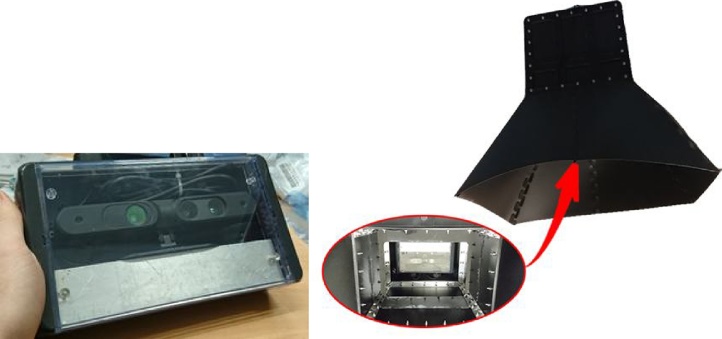
Asus Xtion Pro RGB-D camera, inside an IP68-rated weather-proof box and mounted within a customised shroud for eliminating direct sunlight.

Challenges 1 and 2 were addressed by affixing a structured-light RGB-D camera (Asus Xtion Pro Live) within an IP68-rated weather-proof box, which was mounted within a specially developed rugged shroud, as shown in Fig. 5.

**Fig. 6:**
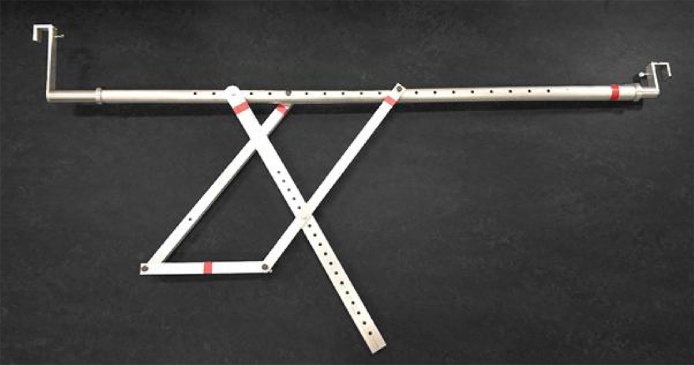
Lightweight adjustable and reconfigurable bracket assemblies, developed for mounting the camera and housing on the Grime GT170 harvester.

While the camera has to be mounted as rigidly as possible in order to withstand the robust environment aboard a harvester, its position also has to be adjustable (to facilitate fitting onto various makes/models of harvester) while not being so heavy as to apply undue loads to the harvester canopy frame (to which it will be attached) during harvesting. Also, to address challenge 3 above, mounting has to be implemented so as to minimise the dynamic effects of the vibrations and movements of the harvester. The solution was to design and manufacture an adjustable and reconfigurable bracket assembly from aluminium alloy, as shown in Fig. 6. Two such assemblies were employed to mount the shroud and camera onto a Grime GT170 harvester that was used for the trials.

**Fig. 7:**
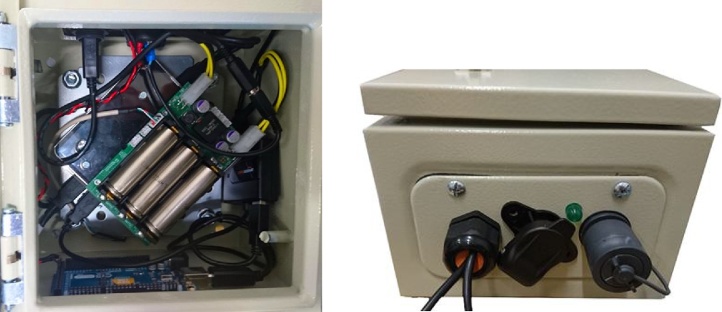
The contents and connections for the Processing Box; the rechargeable batteries of the OpenUPS2 uninterruptible power supply are visible in the left image and the weatherproof USB socket can be seen on the far right.

Waterproofing also involved employment of an IP68 rated protective casing for the ‘Processing Box’, which contained: a small-form computer (Intel Nuc Kit NUC6i5SYK Intel Core i5-6260U Barebone), which acted as the main processor for the device, a USB hub and an OpenUPS2 uninterruptible power supply. The latter is needed to address challenge 4; so that when the tractor powers down, the device shuts down gracefully, rather than having the power cut immediately. The Processing Box also contained the USB hub and a status LED, used to indicate when USB writing is complete (and an Arduino to operate the LED). Fig. 7 shows the contents of the box; and the components on the outside fascia of the box, which include a waterproof gland for the camera and GPS cables, a power D-Socket, status LED and the waterproof USB socket (the last two being used to address challenge 5).

**Fig. 8:**
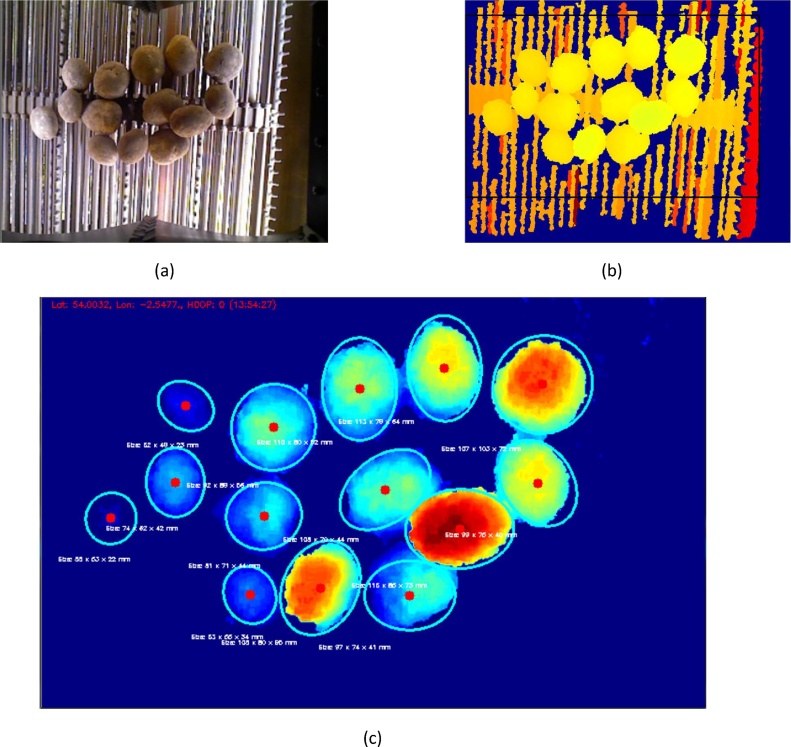
Image of potatoes moving on a conveyor of a harvester, with use of 3D data to facilitate segmentation and measurement of the tubers. (a) The colour frame from the depth camera, (b) The depth information, with the region of interest highlighted (black rectangle), (c) The same depth frame, normalised to remove background and annotated with potato detections and measurements.

Finally, challenge 6 was met by employing a relatively fast shutter speed to ‘freeze’ the potatoes’ motion on the conveyor; and any potential problems due to touching potatoes were overcome by employing the 3D data from the surface of each potato to fit an ellipsoid to it, thereby enabling segmentation. This is shown in Fig. 8 below.

#### 4.2.2 Information generated by the potato metrology system

In order to test the operation of the device, potatoes were measured in daylight in the field, with the harvester moving. The conveyor was started and a number of potatoes of known dimension (length, width and height) were passed along the conveyor, under the device. An example of the results are shown in Table 1, where it can be seen that the majority of measurements obtained are within 10% of their true values.

**Table 1:** Vision system (Est.) measurements and caliper measurements (True) for potatoes moving along a conveyor on a harvester moving through a field. Units are millimetres throughout. Percentage accuracies of each vision system measurement are also given.

ID	True Length	Est. Length	%	True Width	Est. Width	%	True Height	Est. Height	%	True Sz. Square	Est. Sz. Square
**1**	**92**	109	84.4	**78**	60	70.0	**56**	39	56.4	**136**	101
**2**	**92**	116	79.3	**75**	82	91.5	**54**	52	96.2	**131**	137
**3**	**92**	99	92.9	**79**	88	89.8	**54**	52	96.2	**135**	145
**4**	**117**	100	83.0	**93**	88	94.3	**55**	53	96.2	**153**	145
**5**	**102**	107	95.3	**94**	100	94.0	**63**	60	95.0	**160**	165
**6**	**96**	75	72.0	**77**	63	77.8	**58**	33	24.2	**136**	101

Bold indicates measured values.

There is often an over-estimation of the major axis (length), due to potatoes not being true ellipsoids; however, we only use the minor axis (width) and height in our 3D sizing estimations [20], so this does not affect the quality of the resulting size gradings.

**Fig. 9:**
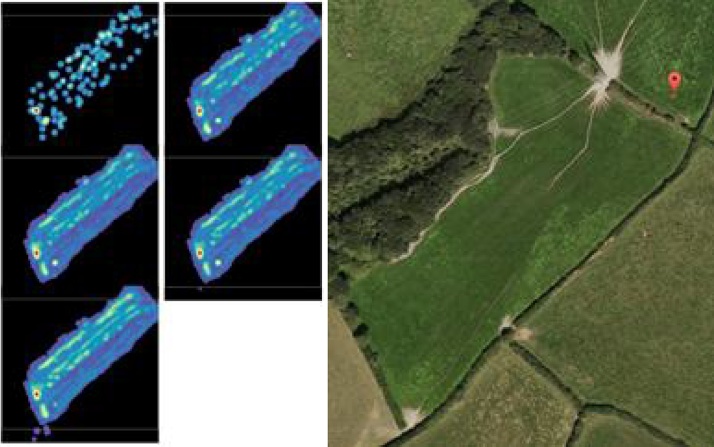
Heat maps (left) and satellite image (right) of a field harvested on 13th July 2016, which give an indication of yield across the field. The colours of the heat maps represent the number of potatoes that have been measured at a particular GPS location. The number of potatoes range from blue (low) to red (high). The five images on the left correspond to five discreet size bands. (For interpretation of the references to colour in this figure legend, the reader is referred to the web version of this article.)

The next stage was to investigate what useful data can be provided by the system when the harvester is working in anger in the field, digging potatoes; where we expect to generate GPS maps that fit neatly with the satellite view of the harvested field. The approach employed here involved measuring how many potatoes were measured to be within each of five discreet size bands, and then displaying this as a heat map. Five heat maps were generated for each field, one showing the yield for each sizing band. The colours of the heat maps represent the number of potatoes within a corresponding band that have been measured at a particular GPS location, where blue indicates a low number of potatoes, and red indicates a large number of potatoes with the corresponding colours in between (i.e. green means a medium number of potatoes were seen). Fig. 9 shows heat maps (left) and satellite image (right) of a field harvested on 13th July 2016.

As can be seen in Fig. 9, the heat maps offer a convenient and potentially powerful way to visualise 3-dimensional data (latitude, longitude, number of potatoes). The potential utility of this is clear; it can enable farmers to easily evaluate yields in particular field (or parts of fields); and to relate this data to factors such as soil type/condition, possible usage of fertilizers and pesticides, as well as environmental conditions and water usage over particular periods. Such information offers much potential for farmers to more fully understand performance factors, thereby assisting them with optimizing yields while reducing/minimising resource usage. This project is believed to be the first to demonstrate the utility of 3D machine vision for segmenting potatoes immediately after being dug up (rather than after being stored for a period), and for determining yield from a field by measuring the number of potatoes and their size distribution. The richer data that can be provided by 3D analysis also allows further information to be generated. By recovering the texture and shape of the potato surface, various characteristics that may be of critical interest, can be detected. These include:

• Presence of disease (e.g. potato blight or fungal infection).
• Damage due to fauna (pests in the soil).
• Damage sustained during harvesting.
• The presence of substantial soil on the potato.

However, effective and reliable identification of such features does require high-resolution recovery of the 3D surface texture of a potato, and one way of achieving this is to apply PS as described above. Application of PS to in-depth potato analysis is a promising area; and it is the subject of on-going research in CMV.

## 5 Discussion

### 5.1 3D machine vision: benefits and data capture techniques

In this paper, the literature review and the outline of machine vision agri-tech work being undertaken in CMV, has identified the potential benefits of the agricultural application of machine vision; and particularly 3D analysis. The specific benefits of the latter can be summarised by the following characteristics of a 3D approach:

• Allows for straightforward segmentation of objects for analysis (as illustrated by the above potato application).
• Can provide accurate surface recovery, including high-resolution 3D texture; with many potential applications, such as introducing new plant measurement capabilities in plant phenotyping.
• Illumination invariant – can still recover useful information in a wide range of lighting conditions, including low light, dark conditions, or even direct sunlight.
• Pose invariant – 3D data can provide objective (object-based) measurements – thereby eliminating problematic effects of camera position that are associated with 2D imaging – such as perspective and parallax.
• Provides additional discriminatory information (such as diseased tubers, damage to tubers sustained during harvesting and soil adhesion).
• Can be used to augment 2D

The example 3D vision agri-tech applications described in this paper illustrate how these benefits can be realised with relatively low-cost vision technologies that are currently emerging. Associated practical considerations and potential advanced capabilities are discussed below.

### 5.2 Detection of meristem for directed weeding; and plant phenotyping

The work detailed in this paper outlines our implementation of 2D and 3D weed analysis methods in order to detect and locate weeds at a field level. CMV has produced a system capable of the detection of weeds in the field and for enabling close analysis of their structure. The system also includes the capability to produce a “field map” by mosaicking frames of video data together then highlighting the weeds upon it. Perhaps most importantly, we have also explored the possibility of using 3D features to determine weed location, both on a static rig and under motion.

Alongside the implemented code, we have produced a highly functional user-interface, pulling together the algorithms into one place. Users can capture, analyse and export data in a variety of modalities and modify parameters to examine their effect on the resulting estimations.

The work investigated both 2D and 3D vision methods for detecting vegetation and localising the meristem of plants. Results were generally good, with many meristems being localised to within a few millimetres of their true location. The 3D data generated from the PS tests are particularly promising – here advanced feature analysis can generate much information for enabling advanced plant analysis capabilities, in both the field and the laboratory. Regarding the latter, a wide range of experiments have been undertaken using the PS apparatus shown in Fig. 3 for accurately monitoring the growth of crops in situ, with detailed measurement of phenotypic properties made possible by the highly detailed surface models we obtain through PS. An example of such a technique is the measurement of plant growth under various soil or fertiliser conditions or at varied levels of irrigation and/or temperature. Work has also included illumination at infra-red wavelengths to reduce the influence of changes in background light (even resulting in reliable data collection in direct sunlight); and with a wide range of light wavelengths (i.e. a multi-spectral approach) with a conventional camera. Also, a hyper-spectral camera (pushbroom type by Gilden Photonics) has been employed for implementing the PS. Plant imaging is well suited to this, since the considerable time that such a camera can take to recover a hyper-spectral image does not present a problem in this application. This is an on-going area of research – our experiments have been very encouraging and this approach promises to offer new advanced capabilities for plant phenotyping – our intention is to present the results of this in an on-going series of journal papers.

### 5.3 Potato measurement during harvesting

A potato metrology system was demonstrated to work well at accuracy levels of within 10% both in the lab and in field trials. Measurements are taken based on a virtual sieving algorithm introduced by Lee [21], which calculates a minimal cross-section of the potato that will pass through a given sizing grid. The system is also able to operate with a relatively wide range of potato sizes (<45, 45–65, 65–80 and 80+ mm). However, a major challenge when implementing a system of this type is how to effectively deal with unexpected problems. Although the work reported here highlighted a number of unforeseen issues, most of these were effectively addressed. An example issue related to the power supply – once the tractor is connected one would have expected a constant supply; but it was found to be very intermittent (lab testing was performed using a 12 V portable battery where this behaviour was not observed). The expectation was that the unit could operate from the tractor power supply, with the UPS only being needed to allow the computer to shut down cleanly when it detected that the line in was disconnected, and also to “clean” the signal from the tractor to produce a reliable 19 V output. However, experience has shown that it would be advisable to run the system from a separate 12 V lead-acid accumulator (i.e. a car battery) and to only employ the tractor power for charging this battery.

To summarise, the potato project has successfully developed and tested a demonstrator of the key technologies and functionalities that would be needed by an eventual production system. As mentioned above, a number of outstanding issues have been identified along the way; other issues worthy of further investigation are a fuller treatment on the effects of foreign materials and of moisture causing increased mud content and specularity in the camera view, and monitoring of tractor speed (possibly through matching of ‘salient’ features in overlapping frames as mentioned above) for interpreting the rate of potato harvesting. The development of any new technology inevitably requires extensive real-wold testing and a process of iterative refinement via, in this case, a program of testing and modification using significant field data capture over an extended period.

## 6 Conclusion

The question a farmer or agri-tech company may wish to ask is: which 3D techniques are most likely to enable the potential benefits (listed in the Discussion) to be realised? From a review of supplier literature it becomes clear that there are a wide range of 3D vision technologies available, each with their particular advantages and drawbacks; examples include: stereo vision, laser triangulation, LIDAR, photometric stereo, and RGB-D cameras. The latter device provides colour information as well as the estimated depth for each pixel. In recent years, RGB-D devices such as the Kinect have become widely available at costs that are around an order of magnitude less than that of previous sensors that had similar functionality. This has resulted in a rapid expansion of their use for 3D machine vision research in various sectors, including agriculture. An example of this is provided by the potato inspection project described above, where the RGB camera provided a cost effective means of deriving the metrics that were of interest to the collaborating company – specifically potato sizes and yields. However, if the emphasis had been more on the individual potato quality characteristics, another method may have been employed to capture surface texture/topology characteristics at high resolution. Photometric stereo (PS) is a technique that is well suited to achieving this, while also having the advantage of being relatively low-cost. The resolution with which PS allows 3D plant textures to be recovered, allows detailed inspection of leaf size/shape and characteristics/condition for implementing cost-effective plant identification and/or crop monitoring. The potential to employ this for widespread realisation of advanced capabilities with long term potential benefits to agriculture, is very great, since the low cost of the needed emerging equipment means that it is very accessible and so can be widely applied. Therefore, with world-wide food security becoming an increasingly urgent matter, plant imaging, and particularly 3D plant analysis, is likely to emerge as an increasingly important technology.

## Competing interests statement

The authors have no competing interests.
